# Correlation between the Oral and Mental Health of University Students in Serbia—A Cross-Sectional Study

**DOI:** 10.3390/healthcare12141431

**Published:** 2024-07-17

**Authors:** Nikoleta Janicijevic, Tamara Dimovic, Dalibor Stajic, Nela Djonovic, Dragan Vasiljevic, Melanija Tepavcevic, Milos Stepovic, Simonida Delic, Marko Petrovic, Kristijan Jovanovic, Ermin Fetahovic, Katarina Manojlovic, Ivica Petrovic, Milos Filipovic, Marija Sekulic

**Affiliations:** 1 Department of Hygiene and Ecology, Faculty of Medical Sciences, University of Kragujevac, 34000 Kragujevac, Serbia; nikoleta.janicijevic@gmail.com (N.J.); stajicdalibor@yahoo.com (D.S.); ndjonovic@medf.kg.ac.rs (N.D.); dvg_gana@yahoo.com (D.V.); msekulic82@gmail.com (M.S.); 2 Doctoral Academic Studies—Medical Sciences, Faculty of Medical Sciences, University of Kragujevac, 34000 Kragujevac, Serbia; milosfilipovic88@hotmail.com; 3 Department of Anatomy, Faculty of Medical Sciences, University of Kragujevac, 34000 Kragujevac, Serbia; melanijatepavcevic@yahoo.com (M.T.); stepovicmilos@yahoo.com (M.S.); simonidastevanovic1996@gmail.com (S.D.); kralj100@yahoo.com (K.J.); 4 Department of Surgery, Faculty of Medical Sciences, University of Kragujevac, 34000 Kragujevac, Serbia; markopetrovickg@yahoo.com; 5 Department of Communication Skills with Information Technologies, Faculty of Medical Sciences, University of Kragujevac, 34000 Kragujevac, Serbia; erminfetahovic96@gmail.com; 6 Department of Physical Medicine and Rehabilitation, Faculty of Medical Sciences, University of Kragujevac, 34000 Kragujevac, Serbia; manojlovickatarina112@gmail.com; 7 Department of Pathophysiology, Faculty of Medical Sciences, University of Kragujevac, 34000 Kragujevac, Serbia; liavaci@gmail.com

**Keywords:** oral health, anxiety, depression, students, Serbia

## Abstract

Background: This cross-sectional study aimed to assess the correlation between subjective oral health status and mental health in a group of university students in Serbia. Methods: The study included 948 students, aged between 18 and 27, from the Faculty of Medical Sciences, University of Kragujevac, Serbia, and was conducted in 2020. The World Health Organization’s Oral Health Questionnaire for Adults was utilized to evaluate the respondents’ self-perceived oral health and oral hygiene practices. The existence of depressive symptoms was evaluated using the Beck Depression Inventory-II (BDI-II), and the Zung self-rating anxiety scale (SAS) was used for determining anxiety symptoms. Results: It is observed that 28.9% of respondents exhibit symptoms of depression and 42.3% showed symptoms of anxiety. Results show that students with severe depression and anxiety symptoms very often reported lower self-perceived oral health and oral health problems. Although the nature of this relationship has not been thoroughly evaluated, several studies have shown a mutually dependent connection between oral health and mental health. Conclusions: The results suggest that some aspects of oral health are associated with higher risks of developing symptoms of depression or anxiety, and vice versa.

## 1. Introduction

Worldwide in 2019, 280 million individuals, including 23 million children and adolescents lived with depression. Anxiety disorders, as one of the most frequent mental disorders, were present in 301 million individuals, including 58 million children and adolescents [1].

It is generally acknowledged that dental health is important for overall health. Both dentistry and medical students are required to be well aware of oral health issues and to promote oral health in the general public [2].

The World Health Organization (WHO) has recently engaged in extensive epidemiological research with the aim of identifying and validating the connection between mental conditions and physical issues.

Individuals with depression or anxiety problems receive dental care far less frequently and have a roughly 30% higher risk of tooth loss than those with good mental health. Notable risk factors for developing oral disorders include depression and anxiety disorders, particularly for bruxism, which can lead to tooth abrasion and overextension, temporomandibular joint disorders (TMDs), and eventually loosening of teeth [2].

Despite compelling evidence supporting the interdependence between mental illnesses and other health conditions, little is known about how dental health affects these conditions. At least half of dental patients are said to experience anxiety before their appointments, and part of them can even develop anxiety disorders. This relationship is still disregarded even though there is growing evidence that people with mental health issues have poor oral health [3].

Medical school environments are extremely demanding, which can lead to high levels of anxiety, among other health issues, as well as having an impact on a student’s academic performance [2].

People who suffer from mental illness are more likely to have oral health issues due to poor nutrition and oral hygiene, excessive consumption of sugary drinks, co-occurring substance abuse (tobacco, alcohol, psychostimulants, etc.), and/or barriers to dental care due to cost or other factors [4].

There is a scarcity of research on the connection between mental health and oral health status among university students in Serbia. This cross-sectional study aimed to analyze the association between subjective oral health status and mental illness in a group of university students in Serbia.

## 2. Materials and Methods

### 2.1. Study Procedure and Participants

Students who were enrolled in the study that was enacted from January to March 2020, attended medical, pharmacy, dentistry, and basic vocational studies at the Faculty of Medical Sciences, University of Kragujevac. Out of 948 participants (age ranging 18–27 years), 48 did not disclose their gender. However, out of those who did, 78% (n = 700) were female students, 22% (n = 200) were male students. Questionnaires were handed out in a paper form after the regular classes/lectures, with prior approval of the board of Faculty of Medical Sciences, University of Kragujevac. Students gave their consent to take part in an anonymous survey. Questions/issues that were not fully answered were excluded from the statistical analysis.

### 2.2. Applied Questionnaires

#### 2.2.1. Socio-Demographic Data

Components of questionnaire: basic information (gender, age); anthropometric information (weight, height); information about eating/drinking habits (consumption of meat, bread, vegetables, fruits, etc.; consumption of beverages) and information about satisfaction with physical appearance, duration and types of physical activity. 

#### 2.2.2. Symptoms of Depression

The BDI-II scale was used to assess symptoms of depression. This scale consists of 21 questions, where each one is graded between 0 and 3 points. Intention was to record a range of depression symptoms the person has encountered throughout the previous week. The rating scale was as follows: 0–13: no/minimal symptoms; 14–19: mild depressive symptoms; 20–28: moderate depressive symptoms; 29–63: severe depressive symptoms [5,6].

#### 2.2.3. Symptoms of Anxiety

The Zung Self-Rating Anxiety Scale (SAS) is a 20-item self-report assessment tool designed to quantify anxiety levels. In response to the claims, a person should answer how much each one applies to them in the one to two weeks leading up to the test. Each query is graded from 0 to 4 using a Likert-type scale (based on these replies: “a little of the time”, “some of the time”, “good part of the time”, “most of the time”). To get around the issue of a set response, certain questions are negatively phrased. A raw score of 36 was the proposed cut-off point in 1980, according to recent studies [7,8,9].

#### 2.2.4. Oral Health

The World Health Organization’s Oral Health Questionnaire for Adults was utilized to evaluate the respondents’ self-perceived oral health, oral health problems, and oral hygiene practices. It includes 16 questions on oral hygiene practices, oral health status, frequency of dental visits, and subjective perception of oral health [10].

The questionnaires were validated and used in the Serbian language [6,9,10].

### 2.3. Statistical Analysis

Statistical analysis was performed using the Statistical Package for Social Sciences software (SPSS Inc., version 19.0, Chicago, IL, USA). Chi-square test was used to determine the relationship between categorical data. We presented only significant results where *p* < 0.05. Phi-coefficient and Cramer’s V were utilized for examining the association between two categorical variables when there are 2 × 2 and more than 2 × 2 contingency tables, respectively. The relationship between dependent variables and a series of independent variables was examined by univariate logistic regression. The risk was evaluated using the OR (odds ratio) size, with a 95% confidence interval. Spearman’s correlation coefficient was used for the correlation of the scales. With a total population of 1566 students when the confidence interval is 95% and the margin error is 5%, the representative sample is calculated to be 309 students. Our study included more students than the calculated representative sample (948 participants).

## 3. Results

Reliability and internal consistency of a scale BDI expressed by Cronbach’s alpha coefficient was assessed as excellent (α = 0.91). The reliability of the scale for anxiety, estimated on the basis of the value of Cronbach’s alpha, was somewhat lower compared to the BDI scale, but still acceptable (0.70). 

The distribution of the values of the scales did not follow the normal distribution (Kolmogorov–Smirnov test, *p* = 0.00 for both scales), so accordingly, Spearman’s correlation coefficient was used for the correlation of the scales. A strong positive correlation was found between these two scales (Spearman’s rho = 0.709).

It is observed that 28.9% of respondents exhibit symptoms of depression and 42.3% showed symptoms of anxiety, see Table 1 and Table 2.

While mild symptoms were present in 14.4%, moderate symptoms in 8.8%, and severe symptoms in 4.7% of respondents, the majority of respondents (72.1%) did not experience any signs of depression. In addition, considerably more female students showed symptoms of severe depression (5%) than male students (3.4%), (Pearson = 11.474, df = 3, *p* = 0.009). However, older participants with severe depression symptoms were reported in a lower percentage (3.7%), compared to their younger counterparts (5.3%).

The relationship between depressive symptoms and the factors that refer to oral health is shown in Table 1. Everyday issues that were very often consequences of poor oral health were discernible and higher in percentage in those with symptoms of severe depression than in those with mild or no symptoms. Students with severe depression reported very often difficulty biting off (4.5%), difficulty speaking (2.3%), dry mouth (4.5%), ashamed of appearance (6.8%), pressure/discomfort (6.8%), avoiding smiling (7.0%), interrupted sleep (9.1%), difficulty in everyday activities (4.5%), and reduced social interaction (6.8%). However, absence from college was solely significant in male respondents (*p* = 0.000), with 57.1% of males with severe depression symptoms stating that they were not absent from work/college due to oral problems. Subjectively, reported conditions of teeth and gum are notably different in male students, where 14.3% of male students with symptoms of severe depression reported bad teeth condition in comparison to 0.0% of those with no or mild symptoms. Very bad gum condition is disclosed in 2.9% of female students who displayed symptoms of severe depression, in contrast to 0.0% without the same symptoms. A higher percentage of students who showed symptoms of severe depression (65.1%) used fluorides than students with fewer depression symptoms.

The relationship between the Zung Self-Rating Anxiety Scale (SAS) and the factors regarding oral health are shown in Table 2. Excellent teeth condition was reported in 36.5% of female students with an anxiety score lower than 36, and 28.4% who showed symptoms of anxiety. As for gum condition, the answer, excellent, was given by 50.6% of female students who showed minimal or no anxiety symptoms, and 37% with anxiety symptoms. Statistically significant were the everyday issues concerning oral health, including difficulty biting off (*p* = 0.009), difficulty chewing food (*p* = 0.007), difficulty speaking (*p* = 0.003), dry mouth (*p* = 0.000), being ashamed of one’s appearance (*p* = 0.000), pressure/discomfort (*p* = 0.000), avoiding smiling (*p* = 0.002), interrupted sleep (*p* = 0.000), difficulty in everyday activities (*p* = 0.000), and reduced social interaction (*p* = 0.021). 

All values of Cramer’s indicator indicate a weak connection between the variables (weak influence according to Cohen’s criteria) (Table 1 and Table 2).

Regression analysis in the total population did not show a significant association of symptoms of depression and anxiety with oral health in students, except for the variables of difficulty in everyday activities, interrupted sleep, and pressure/discomfort in the multivariate model (Table 3). However, the results of logistic regression by gender, related to oral health, showed that female participants, who very often felt pressure and discomfort (OR = 12.548, *p* = 0.01), avoided smiling (OR = 12.968, *p* = 0.006), had difficulties in daily activities (OR = 22.321, *p* = 0.002), and had less social interaction (OR = 14.133, *p* = 0.004), had a significantly higher chance of manifesting more serious symptoms of depression compared to those who answered negatively. Female students who very often felt pain and discomfort (OR = 1.596, *p* = 0.003), used cigarettes (OR = 2.748, *p* = 0.005), had dry mouth (OR = 6.080, *p* = 0.002), felt pressure and discomfort (OR = 8.565, *p* = 0.045), and had sleep interruptions (OR = 15.993, *p* = 0.001) had a statistically significantly higher chance of exhibiting anxiety symptoms compared to those who answered negatively. Women who sometimes had a problem with biting food (OR = 1.888, *p* = 0.012), chewing (OR = 12.985, *p* = 0.000), speaking (OR = 3.716, *p* = 0.001), and difficulties in daily activities (OR = 2.852, *p* = 0.001), also had a statistically significant higher chance of exhibiting symptoms of anxiety compared to those who answered negatively. When it comes to male counterparts, those who very often felt embarrassed (OR = 23.120, *p* = 0.018), avoided smiling (OR = 24.000, *p* = 0.016), had interrupted sleep (OR = 14.167, *p* = 0.006), and had fewer social interactions (OR = 12.000, *p* = 0.043) had a significantly higher chance of exhibiting more serious symptoms of depression compared to male students who answered negatively. Male participants who had the best dental condition (OR = 0.019, *p* = 0.019) had less chance of more serious symptoms of depression. Men who used cigarettes very often (OR = 1.860, *p* = 0.05) and had interrupted sleep (OR = 6.729, *p* = 0.026) were statistically significantly more likely to exhibit symptoms of anxiety compared to those who answered negatively.

## 4. Discussion

Correspondences between mental conditions and dental health are uncovered every year; despite this, the connection is still overlooked.

A meta-analysis by Quek et al. [11] revealed that anxiety was more common in female medical students than in male students, but without a statistically significant difference. Our findings indicate a statistically significant difference between males and females, with females having a higher prevalence of anxiety symptoms.

Studies by Pitułaj et al. and Okoro et al. [3,12] exhibited that participants who had shown anxiety or depression symptoms utilized dental services less frequently; however, in our study, there was no significant difference in those with and without anxiety and depression symptoms regarding the time of the last visit to the dentist.

In comparison to a previous study by Stepovic et al. [13], where no statistically significant difference was found when examining the relationship between gender and the BDI scale, our study showed considerably more female students had shown symptoms of severe depression than male students. Moreover, our study showed no significant difference regarding the correlation between symptoms of depression and the frequency of toothbrushing, contrasting with the above-mentioned study [13]. However, our study showed that 14.3% of male respondents with symptoms of severe depression reported bad teeth condition in comparison to 0.0% of those with no or mild symptoms, which contradicts the findings of related research carried out on Serbian students, which indicated that a greater proportion of male respondents without depressive symptoms had poor oral health than those with symptoms [13].

Smoking is more common in people with anxiety symptoms according to the previous research, which is in agreement with our research. Analysis of the data showed a significant difference in females (30.2%) and males (42.9%) with symptoms of anxiety who reported themselves as smokers [14].

In contrast to our study, where younger students more often reported severe depression (5.3%) and anxiety (48.8%) symptoms, in China, older medical students had a higher prevalence of the above-mentioned symptoms [15].

Although the nature of this relationship has not been thoroughly evaluated, several studies have shown a mutually dependent connection between oral health and mental health [16] and have tried to elucidate possible reasons for this association. The low priority of oral health, poor recognition of the link between poor oral and mental health by healthcare professionals, and the lack of alternative service models in dental facilities for those with increased anxiety and/or mental health problems have been identified as some of the underlying reasons for this link [17].

However, some studies have shown that depression and anxiety have no effect on dental health. They also showed that men have better oral health, and women have better dental care, and that education is associated with better dental care [18].

Individuals with mental illness are more likely to have suboptimal oral health than those without [19].

Depressive symptoms can negatively affect behavior related to oral hygiene and the treatment of dental diseases. A higher risk of tooth decay and tooth loss may lead to more frequent pain, social isolation and low self-esteem, and reduced quality of life, resulting in poorer mental and overall health [20].

Our study has some limitations. The results of the survey are based on a cross-sectional survey, and all data were self-reported by the participants; hence, there were no objective measures of oral health. We cannot conclude that these results apply to students from other faculties because the student population is from the Faculty of Medical Sciences, and it may be presumed that they already have prior knowledge of the evaluated issue. We intend to acquire similar data in the future to maintain the continuity of research and work to better understand the relationship between mental and oral health over time. Despite these limitations, this study offers a significant contribution to the literature, highlighting the association between oral health status and poor mental health at the population level. Further research is necessary to understand the mechanisms underlying these associations.

## 5. Conclusions

A sample of Serbian students was used in our research to examine the relationship between oral health and symptoms of depression and anxiety. 

The study showed a significant correlation between common mental health disorders and oral health. 

Although the findings are inconclusive and somewhat contradictory as to whether oral health affects mental health or vice versa, we can conclude, based on the previous studies and our results, that there is a mutual and bidirectional correlation between oral and mental health. That is why our study can offer an opportunity to consider whether a more integrated approach to oral health and mental health for students, as well as for the rest of the population, is necessary.

## Figures and Tables

**Table 1:** Symptoms of depression associated with oral health by gender and in total.

Variables	0 to 13 No/Minimal			14 to 19 Mild			20 to 28 Moderate			29 to 63 Severe			Pearson Chi-Square/df/*p*/Phi-Cramer		
	*F*	*M*	*T*	*F*	*M*	*T*	*F*	*M*	*T*	*F*	*M*	*T*	*F*	*M*	*T*
N	515	187	674	118	22	135	70	16	82	37	8	44			
%	69.6	80.3	72.1	15.9	9.4	14.4	9.5	6.9	8.8	5	3.4	4.7			
**Fluorides in toothpaste**													12.894/6/ 0.045/ 0.133	3.455/6/ 0.750/ 0.136	13.014/6/ 0.043/ 0.084
yes	55.1	59.5	56.0	60.7	64.7	59.7	51.5	83.3	57.3	65.7	71.4	65.1			
no	2.9	1.9	2.8	0.9	0.0	0.7	9.1	0.0	8.5	0.0	0.0	2.3			
I don’t know	42.0	38.6	41.2	38.4	35.3	39.6	39.4	16.7	34.1	34.3	28.6	32.6			
**Cigarettes**													16.662/3/ 0.001/0.148	5.712/3/ 0.126/0.184	15.968/3/ 0.001/0.133
yes	19.7	28.0	22.5	29.1	53.3	33.1	38.7	41.7	39.7	36.1	14.3	31.8			
no	80.3	72.0	77.5	70.9	46.7	66.9	61.3	58.3	60.3	63.9	85.7	68.2			
**Drinks in the last 30 days**													22.811/9/ 0.007/ 0.090	9.813/9/ 0.366/ 0.156	18.081/9/ 0.034/ 0.075
one	18.9	15.7	17.7	19.5	5.9	17.8	13.6	33.3	17.1	13.9	0.0	13.6			
2 to 3	25.9	27.7	26.7	27.4	35.3	28.9	42.4	33.3	41.5	27.8	28.6	27.3			
4 to 5	11.7	31.4	16.8	18.6	47.1	21.5	10.6	8.3	11.0	30.6	42.9	31.8			
I don’t drink	43.5	25.2	38.8	34.5	11.8	31.9	33.3	25.0	30.5	27.8	28.6	27.3			
**Teeth condition**													18.072/15/0.259/ 0.088	48.033/15/0.000/ 0.247	28.899/15/0.017/ 0.102
excellent	35.9	29.1	33.9	29.2	6.3	26.1	22.7	9.1	22.2	25.0	14.3	22.7			
very good	38.4	44.9	40.7	33.6	31.3	34.3	39.4	63.6	42.0	33.3	14.3	31.8			
good	15.1	18.4	15.5	23.9	18.8	22.4	19.7	27.3	21.0	22.2	42.9	25.0			
average	8.7	7.0	8.1	9.7	25.0	11.9	16.7	0.0	13.6	16.7	14.3	15.9			
bad	1.2	0.0	0.9	2.7	6.3	3.0	1.5	0.0	1.2	2.8	14.3	4.5			
very bad	0.0	0.0	0.0	0.0	0.0	0.0	0.0	0.0	0.0	0.0	0.0	0.0			
I don’t know	0.8	0.6	0.9	0.9	12.5	2.2	0.0	0.0	0.0	0.0	0.0	0.0			
**Gum condition**													33.247/18/0.016/ 0.123	51.338/18/0.00/ 0.224	36.757/18/0.006/ 0.116
excellent	47.2	42.2	45.6	35.1	25.0	33.3	37.5	9.1	35.4	37.1	0.0	32.6			
very good	30.5	35.7	32.3	28.8	31.3	29.5	28.0	27.3	26.6	22.9	42.9	25.6			
good	12.7	14.9	13.0	21.6	18.8	21.2	15.6	63.6	21.5	25.7	28.6	25.6			
average	5.8	4.5	5.6	8.1	12.5	9.1	14.1	0.0	11.4	11.4	28.6	14.0			
bad	2.7	1.9	2.4	4.5	0.0	3.8	4.7	0.0	3.8	0.0	0.0	0.0			
very bad	0.0	0.6	0.2	0.9	0.0	0.8	0.0	0.0	0.00	2.9	0.0	2.3			
I don’t know	1.0	0.0	0.9	0.9	12.5	2.3	0.0	0.0	1.30	0.0	0.0	0.0			
**Difficulty biting off**													22.218/12/0.035/ 0.094	17.989/12/0.116/ 0.139	21.952/12/0.038/ 0.089
very often	1.2	1.3	1.2	0.0	0.0	0.0	1.5	9.1	2.5	5.6	0.0	4.5			
relatively often	1.0	1.3	1.1	2.7	0.0	2.3	1.5	9.1	2.5	0.0	0.0	0.0			
sometimes	10	8.9	9.8	9.8	12.5	10.5	18.2	18.2	17.3	16.7	0.0	13.6			
no	86.5	86.1	86.2	83.0	75.0	82.0	78.8	63.6	77.8	77.8	85.7	79.5			
I don’t know	1.2	2.5	1.8	4.5	12.5	5.3	0.0	0.0	0.0	0.0	14.3	2.3			
**Difficulty speaking**													30.359/12/0.002/ 0.111	15.063/9/0.089/ 0.111	27.459/12/0.007/ 0.100
very often	0.2	0.6	0.3	0.0	0.0	0.0	3.0	0.0	2.5	2.8	0.0	2.3			
relatively often	0.6	0.0	0.6	1.8	0.0	1.5	3.0	0.0	2.5	0.0	0.0	0.0			
sometimes	3.5	4.5	3.8	7.3	6.3	6.9	7.6	18.2	8.6	11.1	0.0	9.1			
no	94.6	94.3	94.1	87.3	87.5	87.8	86.4	81.8	86.4	86.1	85.7	86.4			
I don’t know	1.0	0.6	1.2	3.6	6.3	3.8	0.0	0.0	0.0	0.0	14.3	2.3			
**Dry mouth**													30.052/12/0.003/ 0.117	14.986/12/0.242/ 0.141	33.771/12/0.001/ 0.110
very often	2.5	1.3	2.3	2.7	0.0	2.3	6.1	9.1	6.2	5.6	0.0	4.5			
relatively often	6.0	7.0	6.2	9.0	12.5	9.8	4.5	0.0	3.7	16.7	14.3	15.9			
sometimes	34.8	31	34.3	45.9	37.5	44.7	54.5	36.4	53.1	47.2	14.3	40.9			
no	55.3	59.5	55.8	41.4	43.8	41.7	34.8	54.5	37.0	30.6	57.1	36.4			
I don’t know	1.4	1.3	1.5	0.9	6.3	1.5	0.0	0.0	0.0	0.0	14.3	2.3			
**Ashamed of appearance**													42.907/12/0.000/ 0.142	29.160/12/0.004/ 0.210	54.618/12/0.000/ 0.141
very often	0.4	1.3	0.6	0.9	0.0	0.8	0.0	0.0	0.0	5.6	14.3	6.8			
relatively often	1.7	1.9	1.7	2.7	0.0	2.3	7.6	0.0	6.3	2.8	14.3	4.5			
sometimes	7.1	9	7.5	13.4	12.5	14.3	16.7	30.0	17.5	19.4	14.3	18.2			
no	89.9	87.1	89.5	79.5	81.3	78.9	75.8	70.0	76.3	72.2	42.9	68.2			
I don’t know	0.8	0.6	0.8	3.6	6.3	3.8	0.0	0.0	0.0	0.0	14.3	2.3			
**Pressure/discomfort**													37.521/12/0.000/ 0.127	16.436/12/0.172/ 0.125	33.515/12/0.001/ 0.110
very often	0.6	1.3	0.8	0.9	0.0	0.8	3.0	0.0	2.5	8.3	0.0	6.8			
relatively often	2.3	2.6	2.3	3.6	0.0	3.0	7.6	0.0	6.2	2.8	0.0	2.3			
sometimes	16	19.9	17.1	22.3	12.5	21.1	22.7	45.5	24.7	30.6	14.3	27.3			
no	79.6	75.6	78.5	68.8	81.3	70.7	63.6	54.5	64.2	55.6	71.4	59.1			
I don’t know	1.5	0.6	1.4	4.5	6.3	4.5	3.0	0.0	2.5	2.8	14.3	4.5			
**Avoiding smiling**													23.299/12/0.025/0.105	28.610/12/0.004/0.196	34.950/12/0.000/0.113
very often	0.4	1.3	0.6	0.9	0.0	0.8	0.0	0.0	0.0	5.7	14.3	7.0			
relatively often	1.9	3.2	2.1	0.9	0.0	0.8	4.6	0.0	3.8	5.7	14.3	7.0			
sometimes	7.4	5.1	6.8	9.9	12.5	10.6	10.8	27.3	12.5	2.9	14.3	4.7			
no	88.9	89.2	89.1	85.6	81.3	84.8	84.6	72.7	83.8	85.7	42.9	79.1			
I don’t know	1.5	1.3	1.4	2.7	6.3	3.0	0.0	0.0	0.0	0.0	14.3	2.3			
**Interrupted sleep**													43.597/12/0.000/ 0.135	35.511/12/0.000/ 0.203	47.569/12/0.000/ 0.131
very often	1.7	3.2	2.1	6.3	0.0	5.3	12.1	9.1	11.1	8.3	14.3	9.1			
relatively often	3.1	1.3	2.7	4.5	6.3	5.3	12.1	9.1	11.1	2.8	28.6	6.8			
sometimes	14.6	14.7	14.4	17	6.3	15.8	15.2	9.1	14.8	22.2	0.0	18.2			
no	79.5	79.5	79.5	68.8	75.0	69.2	59.1	72.7	61.7	66.7	42.9	63.6			
I don’t know	1.0	1.3	1.2	3.6	12.5	4.5	1.5	0.0	1.2	0.0	14.3	2.3			
**Absence from work/college**													16.978/12/0.150/ 0.097	35.511/12/0.000/ 0.100	15.544/12/0.213/ 0.080
very often	0.0	1.3	0.3	0.9	6.3	1.5	0.0	0.0	0.0	2.9	0.0	2.3			
relatively often	1.0	0.0	0.8	0.9	0.0	0.8	3.0	0.0	2.5	0.0	0.0	0.0			
sometimes	7.5	7.1	7.3	10.8	12.5	11.4	9.1	27.3	11.1	11.4	28.6	14.0			
no	90.0	90.4	90.3	86.5	75.0	84.8	87.9	72.7	86.4	85.7	57.1	81.4			
I don’t know	1.5	1.3	1.4	0.9	6.3	1.5	0.0	0.0	0.0	0.0	14.3	2.3			
**Difficulty in everyday activities**													45.434/12/0.000/ 0.141	13.690/9/0.134/ 0.135	36.342/12/0.000/ 0.115
very often	0.2	1.3	0.5	0.0	0.0	0.0	1.5	9.1	2.5	5.6	0.0	4.5			
relatively often	0.8	0.0	0.6	1.8	0.0	1.5	3	0.0	2.5	0.0	0.0	0.0			
sometimes	4.8	8.3	5.7	11.6	6.3	10.5	15.2	18.2	14.8	19.4	0.0	15.9			
no	92.5	89.2	91.7	83.9	87.5	85.0	80.3	72.7	80.2	75.0	85.7	77.3			
I don’t know	1.7	1.3	1.5	2.7	6.3	3.0	0.0	0.0	0.0	0.0	14.3	2.3			
**Reduced social interaction**													38.786/12/0.000/ 0.130	45.801/12/0.000/ 0.214	43.999/12/0.000/ 0.126
very often	0.4	1.9	0.8	0.0	0.0	0.0	1.5	9.1	2.5	5.6	14.3	6.8			
relatively often	1.0	0.0	0.9	1.8	0.0	1.5	9.2	0.0	7.5	2.8	14.3	4.5			
sometimes	4.6	6.4	4.8	4.4	18.8	6.7	3.1	18.2	5.0	11.1	14.3	11.4			
no	92.5	90.4	92.0	92	75	89.6	86.2	72.7	85.0	80.6	42.9	75.0			
I don’t know	1.5	1.3	1.5	1.8	6.3	2.2	0.0	0.0	0.0	0.0	14.3	2.3			

**Table 2:** Symptoms of anxiety associated with oral health by gender and in total.

Variables	≤36			>36			Pearson Chi-Square/df/*p*/Phi-Cramer V		
	*F*	*M*	*T*	*F*	*M*	*T*	*F*	*M*	*T*
*N*	362	151	493	348	69	401			
*%*	51	68.6	55.1	49	31.4	44.9			
**Pain, discomfort**							8.392/2/0.015/0.118	0.383/2/0.826/0.029	6.359/2/0.042/0.085
yes	37.5	44.6	38.7	46.7	40.4	45.6			
no	56.6	46.2	54.6	45.5	48.1	46.1			
I don’t know	5.9	9.2	6.7	7.9	11.5	8.3			
**Cigarettes**							12.173/1/0.000/0.123	5.165/1/0.023/0.137	13.141/1/0.00/0.126
yes	18.2	24.0	20.8	30.2	42.9	31.9			
no	81.8	76.0	79.2	69.8	57.1	68.1			
**Drinks in the last 30 days**							11.158/3/0.011/0.119	1.392/3/1.707/0.074	5.688/3/0.128/0.080
one	20.5	17.6	19.3	17.2	11.3	16.5			
2 to 3	22.5	29.0	25.2	31.9	28.3	31.3			
4 to 5	12.0	29.8	16.6	15.1	35.8	18.3			
I don’t drink	45.0	23.7	38.9	35.8	24.5	34.0			
**Teeth condition**							12.978/5/0.024/0.133	13.129/5/0.022/0.236	13.778/5/0.017/0.124
excellent	36.5	30.0	34.8	28.4	11.8	25.9			
very good	39.5	40.8	40.2	36.6	51.0	39.5			
good	13.2	20.0	14.6	21.5	21.6	20.9			
average	9.1	8.5	8.7	10.9	7.8	10.3			
bad	1.5	0.0	1.0	1.5	3.9	1.8			
I don’t know	0.3	0.8	0.6	1.2	3.9	1.5			
**Gum condition**							25.472/6/0.000/0.179	14.702/6/0.023/0.207	26.941/6/0.000/0.175
excellent	50.6	41.4	47.5	37.0	25.5	35.9			
very good	30.5	35.2	31.9	29.3	31.4	30.0			
good	9.2	14.8	10.9	20.4	31.4	21.0			
average	7.1	5.5	6.8	7.4	7.8	7.4			
bad	1.8	2.3	1.9	4.3	0.0	3.6			
very bad	0.3	0.8	0.4	0.3	0.0	0.3			
I don’t know	0.6	0.0	0.6	1.2	3.9	1.8			
**Difficulty biting off**							11.006/4/0.027/0.118	11.971/4/0.018/0.198	13.419/4/0.009/0.123
very often	1.5	0.0	1.0	1.2	3.8	1.5			
relatively often	0.9	1.6	1.0	1.8	1.9	1.8			
sometimes	7.7	6.2	7.4	13.9	17.3	14.1			
no	89.1	89.1	88.7	80.6	71.2	79.8			
I don’t know	0.9	3.1	1.9	2.4	5.8	2.8			
**Difficulty chewing food**							23.394/4/0.000/0.160	3.992/4/0.407/0.122	13.963/4/0.007/0.126
very often	1.2	0.0	0.8	0.6	1.9	0.8			
relatively often	0.6	0.8	0.8	1.8	0.0	1.5			
sometimes	4.7	11.6	6.8	14.1	13.5	13.5			
no	92.6	86.0	90.1	81.0	80.8	81.7			
I don’t know	0.9	1.6	1.4	2.4	3.8	2.5			
**Difficulty speaking**							17.281/4/0.002/0.143	3.000/2/0.223/0.070	15.956/4/0.003/0.135
very often	0.3	0.0	0.2	0.9	0.0	0.8			
relatively often	0.6	0.0	0.6	1.5	0.0	1.3			
sometimes	2.3	4.7	2.9	8.0	7.8	7.7			
no	96.2	94.6	95.3	87.4	88.2	88.0			
I don’t know	0.6	0.8	1.0	2.1	3.9	2.3			
**Dry mouth**							34.891/4/0.000/0.228	3.018/4/0.555/0.130	36.544/4/0.000/0.204
very often	1.2	0.8	1.0	4.3	1.9	4.1			
relatively often	5.9	6.2	6.0	7.6	11.5	8.1			
sometimes	31.2	32.6	31.9	48.2	28.8	45.8			
no	60.6	58.9	59.7	38.7	53.8	40.5			
I don’t know	1.2	1.6	1.4	1.2	3.8	1.5			
**Ashamed of appearance**							20.750/4/0.000/0.175	14.080/4/0.007/0.242	30.496/4/0.000/0.181
very often	0.0	0.0	0.0	1.5	4.1	1.8			
relatively often	0.9	0.8	0.8	3.7	6.1	3.8			
sometimes	7.1	9.4	7.7	12.8	14.3	13			
no	91.4	89.1	90.9	80.1	71.4	79.3			
I don’t know	0.6	0.8	0.6	1.8	4.1	2.0			
**Pressure/discomfort**							21.708/4/0.000/0.161	12.683/4/0.013/0.177	27.239/4/0.000/0.176
very often	0.3	0.0	0.2	2.1	2.0	2.0			
relatively often	2.7	1.6	2.3	3.1	3.9	3.0			
sometimes	14.2	17.2	15.3	22.9	33.3	23.7			
no	82	80.5	81.2	68.2	56.9	67.7			
I don’t know	0.9	0.8	1.0	3.7	3.9	3.6			
**Avoiding smiling**							9.921/4/0.042/0.120	14.761/4/0.005/0.238	16.760/4/0.002/0.139
very often	0.0	0.8	0.2	1.2	1.9	1.3			
relatively often	1.2	1.6	1.2	3.1	7.7	3.6			
sometimes	6.8	4.7	6.2	9	13.5	9.5			
no	91.1	92.2	91.5	84.8	71.2	83.3			
I don’t know	0.9	0.8	0.8	1.9	5.8	2.3			
**Interrupted sleep**							31.727/4/0.00/0.213	15.121/4/0.004/0.244	42.189/4/0.000/0.219
very often	0.6	1.6	0.8	7.4	8.0	7.4			
relatively often	2.9	1.6	2.7	5.8	6.0	5.9			
sometimes	13.5	13.2	13.3	18.4	1.0	17.1			
no	82.1	82.9	82.1	66.6	66.0	67			
I don’t know	0.9	0.8	1.0	1.8	8.0	2.6			
**Difficulty in everyday activities**							22.402/4/0.000/0.169	6.289/3/0.098/0.157	22.155/4/0.000/0.159
very often	0.0	0.0	0.0	1.2	3.9	1.5			
relatively often	0.6	0.0	0.4	1.2	0.0	1.0			
sometimes	4.1	8.5	5.5	11.9	9.8	11.2			
no	94.4	89.9	93.0	83.2	82.4	83.8			
I don’t know	0.9	1.6	1.0	2.4	3.9	2.5			
**Reduced social interaction**							8.020/4/0.091/0.095	10.016/4/0.0400/0.174	11.528/4/0.021/0.115
very often	0.3	0.8	0.4	1.2	5.9	1.8			
relatively often	0.6	0.0	0.6	3.1	2.0	2.8			
sometimes	4.7	6.3	4.9	4.9	11.8	5.9			
no	93.2	91.4	92.6	89.3	76.5	87.8			
I don’t know	1.2	1.6	1.4	1.5	3.9	1.8			

**Table 3:** Regression analysis of depressive and anxiety symptoms associated with oral health in university students.

Variables	BDI ≥ 29		Anxiety Score > 36	
	OR (95% CI)	*p*	OR (95% CI)	*p*
**Pain, discomfort**				
yes	0.742 (0.267–2.059)	0.566	0.953 (0.564–1.608)	0.856
no	0.429 (0.151–1.218)	0.112	0.683 (0.407–1.146)	0.149
I don’t know	1		1	
**Cigarettes use**				
yes	1.608 (0.830–3.113)	0.159	1.783 (1.311–2.426)	<0.001
no	1		1	
**Teeth condition**				
excellent	0.510 (0.159–1.108)	0.958	0.301 (0.074–1.230)	0.095
very good	0.461 (0.092–0.644)	0.745	0.396 (0.098–1.610)	0.196
good	0.551 (0.295–0.826)	0.353	0.576 (0.139–2.388)	0.447
average	0.337 (0.236–0.910)	0.249	0.477 (0.112–2.033)	0.317
bad	1		1	
**Gum condition**				
excellent	0.389 (0.133–0.655)	0.617	0.260 (0.066–1.021)	0.054
very good	0.425 (0.099–0.623)	0.712	0.324 (0.082–1.278)	0.107
good	0.364 (0.169–0.702)	0.534	0.663 (0.164–2.678)	0.564
average	0.304 (0.250–0.804)	0.376	0.377 (0.089–1.592)	0.184
bad	0.420 (0.175–0.655)	0.894	0.667 (0.136–3.272)	0.617
very bad	1		1	
**Difficulty biting off**				
very often	1.099 (0.388–2.485)	0.401	0.982 (0.224–4.305)	0.981
relatively often	0.619 (0.258–1.515)	0.995	1.145 (0.270–4.867)	0.854
sometimes	1.108 (0.122–3.045)	0.928	1.273 (0.480–3.375)	0.628
no	1		1	
**Difficulty chewing food**				
very often	0.556 (0.154–0.865)	0.670	0.525 (0.088–2.118)	0.478
relatively often	0.531 (0.129–0.790)	0.701	1.050 (0.214–3.158)	0.952
sometimes	0.302 (0.136–0.575)	0.542	1.124 (0.390–3.242)	0.828
no	1		1	
**Difficulty speaking**				
very often	1.620 (0.732–4.000)	0.392	1.667 (0.135–4.578)	0.690
relatively often	1.061 (0.573–3.565)	0.996	0.926 (0.153–3.608)	0.933
sometimes	1.280 (0.124–3.175)	0.836	1.190 (0.336–4.214)	0.787
no	1		1	
**Dry mouth**				
very often	1.333 (0.106–3.743)	0.824	1.733 (0.848–4.439)	0.082
relatively often	1.707 (0.188–4.507)	0.635	1.287 (0.387–4.227)	0.680
sometimes	0.789 (0.096–3.518)	0.826	1.362 (0.448–4.139)	0.586
no	1		1	
**Ashamed of appearance**				
very often	0.777 (0.351–2.943)	0.061	0.981 (0.677–2.099)	0.052
relatively often	0.984 (0.055–2.792)	0.991	1.406 (0.250–3.896)	0.699
sometimes	0.859 (0.067–2.103)	0.907	0.517 (0.128–2.081)	0.353
no	1		1	
**Pressure/discomfort**				
very often	1.070 (0.332–2.700)	0.353	0.242 (0.086–0.679)	0.007
relatively often	1.295 (0.318–3.799)	0.300	0.390 (0.105–1.442)	0.158
sometimes	0.478 (0.092–2.473)	0.379	0.449 (0.155–1.303)	0.141
no	1		1	
**Avoiding smiling**				
very often	0.675 (0.191–1.865)	0.142	0.326 (0.099–1.067)	0.064
relatively often	0.929 (0.173–2.154)	0.594	0.799 (0.192–2.222)	0.523
sometimes	0.400 (0.033–0.897)	0.473	1.037 (0.227–3.278)	0.963
no	1		1	
**Interrupted sleep**				
very often	1.203 (0.216–2.868)	0.492	0.328 (0.111–0.969)	0.044
relatively often	1.333 (0.288–3.531)	0.815	0.810 (0.625–3.220)	0.092
sometimes	0.674 (0.075–2.083)	0.725	0.885 (0.248–3.153)	0.850
no	0.427 (0.052–1.531)	0.430	0.515 (0.167–1.590)	0.249
I don’t know	1		1	
**Difficulty in everyday activities**				
very often	0.667 (0.187–2.667)	0.172	0.693 (0.248–1.602)	0.206
relatively often	0.332 (0.045–0.921)	0.996	0.975 (0.303–1.952)	<0.001
sometimes	0.842 (0.202–3.759)	0.588	0.815 (0.251–2.640)	0.733
no	1		1	
**Reduced social interaction**				
very often	1.278 (0.490–3.452)	0.161	0.964 (0.253–2.313)	0.194
relatively often	1.329 (0.246–4.510)	0.365	1.667 (0.703–3.912)	0.123
sometimes	1.562 (0.163–4.995)	0.699	0.958 (0.291–3.161)	0.609
no	1		1	

## Data Availability

The data presented in this study are not available due to privacy.

## References

[1] World Health Organization https://www.who.int/news-room/fact-sheets/detail/mental-disorders.

[2] Yao K., Yao Y., Shen X., Lu C., Guo Q. (2019). Assessment of the oral health behavior, knowledge and status among dental and medical undergraduate students: A cross-sectional study. BMC Oral Health.

[3] Pitułaj A., Kiejna A., Dominiak M. (2019). Negative synergy of mental disorders and oral diseases versus general health. Dent. Med. Probl..

[4] Kisely S. (2016). No Mental Health without Oral Health. Can. J. Psychiatry.

[5] Beck A.T., Steer R.A. (1993). Manual for the Beck Depression Inventory.

[6] Beck A.T., Steer R.A., Brown G.K. (2019). Bekov Inventar Depresije—Drugo Izdanje [Beck Depression Inventory—Second Edition]: BDI-II: Prevod 2. Izdanja: Priručnik. Tom 7.

[7] Zung W.W.K. (1971). A rating instrument for anxiety disorders. Psychosomatics.

[8] Zung W.W.K. (1980). How Normal Is Anxiety?.

[9] Ostojic P., Zivojinovic S., Reza T., Damjanov N. (2010). Symptoms of depression and anxiety in Serbian patients with systemic sclerosis: Impact of disease severity and socioeconomic factors. Mod. Rheumatol..

[10] Lekić M., Daković D., Lazić Z., Čutović T., Ilić I., Ilić M. (2021). Srpska verzija “Upitnika za procenu oralnog zdravlja odraslih”. Vojnosanit. Pregled..

[11] Quek T.T., Tam W.W., Tran B.X., Zhang M., Zhang Z., Ho C.S., Ho R.C. (2019). The Global Prevalence of Anxiety Among Medical Students: A Meta-Analysis. Int. J. Environ. Res. Public Health.

[12] Okoro C.A., Strine T.W., Eke P.I., Dhingra S.S., Balluz L.S. (2012). The association between depression and anxiety and use of oral health services and tooth loss. Community Dent. Oral Epidemiol..

[13] Stepovic M., Rajkovic Z., Stajic D., Sekulic M., Maricic M., Đonović N. (2020). Association between depression levels and self-perceived oral health among medical students. Acta Fac. Medicae Naissensis.

[14] Torales J., Barrios I., González I. (2017). Oral and dental health issues in people with mental disorders. Medwave.

[15] Shao R., He P., Ling B., Tan L., Xu L., Hou Y., Kong L., Yang Y. (2020). Prevalence of depression and anxiety and correlations between depression, anxiety, family functioning, social support and coping styles among Chinese medical students. BMC Psychol..

[16] Kisely S., Baghaie H., Lalloo R., Siskind D., Johnson N.W. (2015). A systematic review and meta-analysis of the association between poor oral health and severe mental illness. Psychosom. Med..

[17] Asif S.M., Assiri K.I., Al Muburak H.M., Baig F.A.H., Arem S.A., Arora S., Shamsudeen S.M., Shariff M., Shamsuddin S., Lahiq A.A.M. (2022). Anxiety and Depression Among Dentists in the Kingdom of Saudi Arabia. Risk Manag. Healthc. Policy.

[18] Scrine C., Durey A., Slack-Smith L. (2018). Enhancing oral health for better mental health: Exploring the views of mental health professionals. Int. J. Ment. Health Nurs..

[19] Solis A.C.O., Marques A.H., Dominguez W.V., Prado E.B.A., Pannuti C.M., Lotufo R.F.M., Lotufo-Neto F. (2016). Evaluation of periodontitis in hospital outpatients with major depressive disorder. A focus on gingival and circulating cytokines. Brain Behav. Immun..

[20] Tiwari T., Kelly A., Randall C.L., Tranby E., Franstve-Hawley J. (2022). Association between Mental Health and Oral Health Status and Care Utilization. Front. Oral. Health.

